# Analysis of postoperative complications in bladder cancer patients

**DOI:** 10.1515/med-2024-1069

**Published:** 2024-11-22

**Authors:** Tianli Shi, Dongdong Yu, Yang Xu, Xiaohui Huang

**Affiliations:** Department of Urology, Huzhou Central Hospital, Fifth School of Clinical Medicine of Zhejiang Chinese Medical University, Huzhou, Zhejiang, 313000, China; Department of Urology, Huzhou Central Hospital, Fifth School of Clinical Medicine of Zhejiang Chinese Medical University, No. 1558, Sanhuan North Road, Wuxing District, Huzhou, Zhejiang, 313000, China

**Keywords:** bladder cancer, postoperative complication, prognosis, surgery, correlation

## Abstract

**Background:**

Bladder cancer, a significant health concern worldwide, often necessitates diverse surgical interventions and postoperative treatments. Understanding the complications arising from these procedures is vital for enhancing patient outcomes and quality of life.

**Methods:**

This study encompassed 80 bladder cancer patients, evaluating their demographic characteristics, systemic conditions, cancer stages, tumor diameter, surgical procedures, and postoperative treatments. The occurrences and types of complications were meticulously documented, alongside the duration and clinical outcomes of these complications. Different surgical procedures were analyzed to discern their respective complication rates.

**Results:**

In all 80 patients, infections (43.75%) emerged as the most common, followed by bladder spasms (16.25%). Notably, complications varied among different surgical procedures, with infection, bladder spasms, and bleeding being prominent in various cases. The correlation analysis did not demonstrate correlation (*r* = 0.13, *p* = 0.26) between bladder cancer stage and duration of complication. Post-treatment interventions, especially anti-infection therapies, showcased positive results, with the majority of patients maintaining or improving their condition after specific treatments.

**Conclusion:**

Our study underscores the diverse landscape of postoperative complications in bladder cancer patients. The findings emphasize the importance of tailored interventions based on specific complications, cancer stages, and surgical procedures.

## 1 Introduction

Bladder cancer is a malignant condition characterized by the uncontrolled growth of abnormal cells within bladder tissues [1]. It ranks among the top ten cancers globally and accounts for a substantial burden in terms of both morbidity and mortality, making it a considerable public health challenge [2].

The most common and noticeable sign of bladder cancer is blood in the urine, also known as hematuria, which is a primary indicator and can be painless or accompanied by discomfort during urination [3]. Other symptoms include changes in urinary habits of increased frequency and urgency; pelvic pain and back pain, especially if the cancer has spread to nearby tissues and organs; and general symptoms like unexplained weight loss and fatigue in advanced stages [4,5]. Several risk factors like smoking, chronic bladder infection, family history, and exposure to chemicals may contribute to the development of bladder cancer [6].

The diagnosis of bladder cancer involves a combination of clinical evaluation, imaging studies, and invasive procedures like cystoscopy and biopsy [7]. Treatment strategies include surgery, radiation therapy, chemotherapy, immunotherapy, and targeted therapy [8]. Among these, surgical resection is a common approach. Current surgical options include transurethral resection of bladder tumor (TURBT), partial cystectomy, radical cystectomy, and robot-assisted laparoscopic cystectomy [9,10]. The choice of surgery depends on the stage and location of the cancer, as well as the patient’s overall health [11]. For early-stage bladder cancer treated with surgery, the prognosis is generally favorable, with a high chance of cure and a low risk of recurrence [12]. However, in cases where the cancer has advanced or spread to other parts of the body, the prognosis may be less optimistic, and additional treatments like chemotherapy or immunotherapy might be necessary to manage the disease and improve outcomes [13]. Regular follow-up appointments and medical monitoring are crucial to detect any potential recurrence early and ensure timely intervention [14].

In addition to the direct impact of surgical outcomes on bladder cancer prognosis, postoperative complications can also affect a patient’s overall outlook [15]. Surgical site infections typically occur within a few days after surgery and are among the most common postoperative complications [16]. Patients may experience redness, swelling, pain, and localized warmth at the surgical site [17]. Treatment usually involves antibiotics or antifungal medications, wound care, and drainage [18]. Hemorrhage can be associated with the surgical incision or coagulation abnormalities after surgery [19]. Continuous or increasing bleeding postoperatively, which may present as hematuria or significant abdominal pain, and treatment may involve blood transfusions, hemostatic medications, repair of the bleeding source, or surgical re-intervention [20,21]. Urethral strictures typically develop within months after surgery, especially in patients who have undergone urethral diversion procedures [22]. Common phenomenon includes difficulty in voiding urine, weak urine flow, urinary dribbling, and possible urgency. Treatment may involve urethral dilation or urethrotomy to widen the narrowed urethra to improve urinary flow and enhance the patient’s quality of life [23].

Postoperative complications have a profound impact on bladder cancer patients. A retrospective study involving 766 patients who underwent radical cystectomy for bladder cancer indicated that patients with a higher comprehensive complication index had a relatively lower overall survival rate and disease-free survival period [24]. Another study involving 147 patients revealed that a high incidence of postoperative complications significantly increased patients’ medical costs and mortality rates [25]. The prognosis of postoperative complications depends on various factors, including timely intervention, the patient’s overall health, and the severity of the complications [26]. Timely diagnosis, treatment, and appropriate rehabilitation plans can significantly improve patient prognosis and mitigate the impact of complications on their lives [27]. This is a critical aspect of our research, which aims to comprehensively understand the occurrence and impact of postoperative complications in bladder cancer patients to guide clinical practice and enhance treatment outcomes [28].

Therefore, our study aims to analyze the incidence, types, clinical characteristics, and outcomes of postoperative complications in bladder cancer patients who have undergone surgical treatment based on clinical data. By investigating these complications comprehensively, we seek to provide insights into their clinical significance, contribute to the enhancement of patient care, and guide future research efforts.

## 2 Methods

### 2.1 Study design

A total of 80 subjects were enrolled in this study.

### 2.2 Subjects

All enrolled subjects were diagnosed with postoperative complications in bladder cancer. A total of 80 individuals included 66 (82.50%) males and 14 (17.50%) females, with a mean age of 68.34 ± 1.36 years. Individuals with systemic conditions included 45 (56.25%) hypertension, 15 (28.75%) smoking, and 23 (28.75%) diabetes mellitus. Postoperative complications mainly focused on infection and bladder spasms, with the percentage of 43.75% (35 cases) and 16.25% (13 cases), respectively. Other complications, including bleeding, gastrointestinal dysfunction, ileus, lower extremity deep vein thrombosis, abnormal liver function, cardiovascular and cerebrovascular abnormalities, and respiratory disease, ranged from 1.25 to 12.50%. Additionally, 8 patients (10.00%) did not exhibit any complications. The average duration of complications was 2.89 ± 0.25 days, ranging from 1 to 12 days.

### 2.3 Statistical analysis

All statistical analyses were performed by using the Statistical Package for Social Sciences (SPSS) software version 26 (IBM Corporation, Armonk, NY, USA). Data were presented as mean ± standard deviation for continuous variables and frequencies (percentages) for categorical variables. The Student’s *t*-test or Wilcoxon rank sum test was used for continuous data. The Chi-square test or Fisher exact test was used to compare categorical variables. A two-tailed *P* value of less than 0.05 was considered statistically significant.


**Informed consent:** The subjects provided written informed consent before participating.
**Ethical approval:** This clinical research was carried out in the Urology Department of Huzhou Central Hospital from January 2021 to December 2022, in accordance with the tenets of the Declaration of Helsinki. Ethical approval was achieved by the Institutional Review Board of Huzhou Central Hospital.

## 3 Results

A total of 80 subjects were included in the study. The demographic characteristics are presented in Table 1. The mean age was 68.34 years with 66 (82.50%) males and 14 (17.50%) females. Several systemic conditions were observed, including 45 (56.25%) hypertension, 15 (28.75%) smokers, and 23 (28.75%) diabetes mellitus. The distribution of bladder cancer stages included Ta in 14 (17.50%) patients, T1 in 43 (53.75%), T2 in 14 (17.50%), T3 in 5 (6.25%), and T4 in 4 (5.00%) patients. The mean tumor diameter was 2.42 cm, ranging from 0.40 to 10.00 cm. Various surgical procedures were performed, including 59 (73.75%) TURBT, 3 (3.75%) laparoscopic partial cystectomy, 8 (1.0%) radical cystectomy with ureterocutaneostomy, 10 (12.50%), and radical cystectomy with ileal neobladder. Postoperative chemotherapy was administered to the majority of patients with 62 (77.50%) receiving it, among which systemic chemotherapy was administered to 2 (2.50%) patients, and intracavitary chemotherapy to 60 (75.00%) patients.

**Table 1:** Demographic characteristics

Variables	*N* = 80
Age (years)	68.34 ± 1.36 (32–90)
Gender	
Male	66 (82.500%)
Female	14 (17.5%)
Systemic condition	
Hypertension	45 (56.25%)
Smoking	15 (28.75%)
Diabetes mellitus	23 (28.75%)
Bladder cancer stage	
Ta	14 (17.50%)
T1	43 (53.75%)
T2	14 (17.50%)
T3	5 (6.25%)
T4	4 (5.00%)
Tumor diameter (cm)	2.42 ± 0.17 (0.4–10.0)
Surgical procedure	
TURBT	59 (73.75%)
Laparoscopic partial cystectomy	3 (3.75%)
Radical cystectomy with ureterocutaneostomy	8 (10.00%)
Radical cystectomy with ileal neobladder	10 (12.50%)
Postoperative chemotherapy	
Yes	62 (77.50%)
No	18 (22.50%)
Chemotherapy method	
Systemic chemotherapy	2 (2.50%)
Intracavitary chemotherapy	60 (75.00%)

Table 2 demonstrates that the most common complication was infection, occurring in 35 subjects (43.75%), followed by bladder spasms in 13 subjects (16.25%). Other complications, including bleeding, gastrointestinal dysfunction, ileus, LEDVT, abnormal liver function, cardiovascular and cerebrovascular abnormalities, and respiratory disease, ranged from 1.25% to 12.50%. Additionally, eight patients (10.00%) did not exhibit any complications. The average duration of complications was 2.89 ± 0.25 days, ranging from 1 to 12 days. Regarding the clinical outcomes of those complications, data indicated that 79 subjects (98.75%) showed improvement and 1 subject (1.25%) maintained their condition.

**Table 2:** Characteristics of postoperative complications

Complication	
Infection	35 (43.75%)
Ileus	1 (1.25%)
Bleeding	10 (12.50%)
Gastrointestinal dysfunction	6 (7.50%)
Bladder spasms	13 (16.25%)
Abnormal liver function	1 (1.25%)
Cardiovascular and cerebrovascular abnormalities	2 (2.50%)
LEDVT	1 (1.25%)
Respiratory disease	2 (2.50%)
None	8 (10.00%)
Duration (days)	2.89 ± 0.25 (1–12)
Outcome	
Improvement	79 (98.75%)
Maintenance	1 (1.25%)

Then, we further analyzed the distribution of complications among patients undergoing different surgical procedures, along with their respective occurrence rates. As shown in Table 3, in patients who underwent TURBT, the most common complications were infection (35.00%), bladder spasms (15.00%), and bleeding (11.25%). For patients receiving radical cystectomy with ureterocutaneostomy, the primary complications included infection (3.75%), ileus (1.25%), and gastrointestinal dysfunction (1.25%). In patients undergoing radical cystectomy with ileal neobladder, the leading complications were infection (5.00%), cardiovascular and cerebrovascular abnormalities (2.50%), and bleeding (1.25%). For those laparoscopic partial cystectomy, the major complications included bladder spasms (1.25%), respiratory disease (1.25%), and gastrointestinal dysfunction (1.25%).

**Table 3:** Top three postoperative complications

	*N* = 80, *n* (%)
TURBT	
Infection	28 (35.00%)
Bladder spasms	12 (15.00%)
Bleeding	9 (11.25%)
Radical cystectomy with ureterocutaneostomy	
Infection	3 (3.75%)
Ileus	1 (1.25%)
Gastrointestinal dysfunction	1 (1.25%)
Radical cystectomy with ileal neobladder	
Infection	4 (5.00%)
Cardiovascular and cerebrovascular abnormalities	2 (2.50%)
Bleeding	1 (1.25%)
Laparoscopic partial cystectomy	
Bladder spasms	1 (1.25%)
Respiratory disease	1 (1.25%)
Gastrointestinal dysfunction	1 (1.25%)

We also conducted a study to explore the relationship between the bladder cancer stage and the prognosis of those complications. We used the Pearson correlation coefficient (*r*) to measure the association between these two variables. As shown in Table 4, the results revealed a significant negative correlation between bladder cancer stage and complication duration (*r* = 0.13, *p* = 0.26), which indicated that the worsening of bladder cancer stage was not associated with the duration of complication.

**Table 4:** Correlation between bladder cancer stage and complication prognosis

*r*	0.13
*P*	0.26

In terms of various treatments in the outcome, we found that the majority of patients showed improvement after interventions as shown in Table 5. In detail, analgesics became the most widely used therapy, which was applied in a total of 22 patients (27.50%). Anti-infection treatment is also a priority choice. Twenty-two patients received antibiotics, and only one patient did not improve. Only one patient underwent a second surgery due to ineffective conservative treatment for bleeding. Other interventions like nutritional support, liver protection, and gastrointestinal therapy along with hemostasis also demonstrated disease relief to some extent.

**Table 5:** Outcome of complication based on treatment

Outcome	Maintenance	Improvement	Total
Analgesic	0	22	22
Nutritional support	0	2	2
Anti-infection	1	21	22
Reoperation	0	1	1
Liver protection	0	1	1
Hemostatic drugs	0	2	2
Gastrointestinal drugs	0	5	5
None	0	25	25

## 4 Discussion

First, the high incidence of infections (43.75%) highlights the significance of stringent perioperative care and infection prevention strategies. This necessitates meticulous preoperative patient evaluation, proper sterilization techniques, and tailored antibiotic prophylaxis to mitigate the risk of infections. There is no relationship between the stage of bladder cancer and the duration of complications (*r* = 0.13, *p* = 0.26), which may be related to the choice of treatment methods for patients in different stages and the skills of surgeons.

The variation in complication rates among different surgical procedures provides valuable guidance for surgeons and clinicians. Research indicates that using laparoscopic or robot-assisted radical cystectomy significantly reduces the occurrence of postoperative complications compared to open radical cystectomy [29,30]. For instance, the relatively lower incidence of complications following radical cystectomy with ureterocutaneostomy suggests that this procedure might be associated with better postoperative outcomes compared to ileal neobladder. This finding advocates for the consideration of ureterocutaneostomy whenever clinically feasible, aiming to balance the risk of postoperative complications and long-term quality of life. Additionally, the dominance of infection and bladder spasms across various surgeries emphasizes the need for targeted preventive measures and vigilant postoperative monitoring for these complications.

Furthermore, the effectiveness of specific interventions, such as anti-infection treatments and reoperation, points toward the importance of individualized patient care. Zhong and Wang observed that applying advanced wound healing techniques, such as negative pressure wound therapy and tissue engineering products, to the surgical incisions after bladder cancer surgery significantly reduces the occurrence of complications [31]. Tailoring interventions based on the type and severity of complications can significantly impact patient recovery. Research efforts could further delve into optimizing these interventions and exploring innovative therapies or techniques to enhance their efficacy.

In a broader context, this study’s results contribute to the growing body of evidence supporting the concept of personalized medicine in oncology. The data underscore the heterogeneity of complications within bladder cancer patients, necessitating customized approaches that consider individual patient profiles, including comorbidities, tumor characteristics, and surgical history. Incorporating advanced technologies like genomic profiling and artificial intelligence in predicting complication risks and treatment responses could revolutionize personalized care, ultimately improving patient outcomes and reducing healthcare costs.

Despite the valuable insights provided by this study, it is essential to acknowledge its limitations, which impact the generalizability and applicability of the findings. One notable limitation is the study’s retrospective nature. Retrospective studies inherently rely on past data, which might be subject to recall bias and incomplete records. This could lead to inaccuracies in patient histories, potentially affecting the analysis of complications and their outcomes. Furthermore, due to geographical and temporal constraints, this study included a relatively small sample size, which may have resulted in selection bias in terms of geographical and age-related factors in patient recruitment. Another limitation pertains to the focus on short-term postoperative complications. Understanding the long-term repercussions of bladder cancer surgery is crucial for holistic patient care and could guide the development of comprehensive rehabilitation programs. In the future, we will continue to recruit a larger number of patients and focus on the association between short-term and long-term complications with patient quality of life and long-term prognosis, aiming to gain a more comprehensive understanding of the impact of postoperative complications on bladder cancer patients.

The comprehensive analysis of postoperative complications in bladder cancer patients provides valuable insights into the complex interplay of factors affecting their outcomes. Beyond the presented data, a deeper exploration of these findings can shed light on the implications for clinical practice and future research.

## 5 Conclusion

This study is based on an in-depth investigation of postoperative complications in bladder cancer patients. It observed different types of complications following surgery, the incidence rates of complications for different surgical procedures, and the differences in patient recovery based on whether interventions were implemented. By integrating these findings into clinical practice and fostering further research collaborations, the medical community can work toward enhancing the quality of care and life for bladder cancer patients, setting a promising direction for the future of oncological treatment.

## References

[1] Dyrskjøt L, Hansel DE, Efstathiou JA, Knowles MA. Bladder cancer. Nat Rev Dis Primers. 2023 Oct;9(1):58. 10.1038/s41572-023-00468-9. PMC11218610 37884563

[2] Richters A, Aben K, Kiemeney L. The global burden of urinary bladder cancer: an update. World J Urol. 2020;38(8):1895–904. 10.1007/s00345-019-02984-4. PMC7363726 31676912

[3] Comperat E, Amin MB, Cathomas R, Choudhury A, De Santis M, Kamat A, et al. Current best practice for bladder cancer: a narrative review of diagnostics and treatments. Lancet. 2022;400(10364):1712–21. 10.1016/S0140-6736(22)01188-6. 36174585

[4] Ahmadi H, Duddalwar V, Daneshmand S. Diagnosis and staging of bladder cancer. Hematol Oncol Clin. 2021;35(3):531–41. 10.1016/j.hoc.2021.02.004. 33958149

[5] Farling KB. Bladder cancer: Risk factors, diagnosis, and management. Nurse Pract. 2017;42(3):26–33. 10.1097/01.NPR.0000512251.61454.5c. 28169964

[6] Jubber I, Ong S, Bukavina L, Black PC, Comperat E, Kamat AM, et al. Epidemiology of bladder cancer in 2023: A systematic review of risk factors. Eur Urol. 2023;84(2):176–90. 10.1016/j.eururo.2023.03.029. 37198015

[7] Sun M, Trinh QD. Diagnosis and staging of bladder cancer. Hematol Oncol Clin. 2015;29(2):205–18. 10.1016/j.hoc.2014.10.013. 25836929

[8] Crabb SJ, Douglas J. The latest treatment options for bladder cancer. Brit Med Bull. 2018;128(1):85–95. 10.1093/bmb/ldy034. 30371744

[9] Comperat E, Oszwald A, Wasinger G, Hansel DE, Montironi R, van der Kwast T, et al. Updated pathology reporting standards for bladder cancer: biopsies, transurethral resections and radical cystectomies. World J Urol. 2022;40(4):915–27. 10.1007/s00345-021-03831-1. PMC8994708 34554298

[10] Ebner B, Eismann L, Volz Y, Bischoff R, Stief CG, Schulz GB. Surgical and systemic therapy of bladder cancer. MMW Fortschr Med. 2022;164(12):40–2. 10.1007/s15006-022-1138-y. 35731405

[11] Pearce S, Daneshmand S. Enhanced endoscopy in bladder cancer. Curr Urol Rep. 2018;19(10):84. 10.1007/s11934-018-0833-9. 30116985

[12] Tohi Y, Miyauchi Y, Yamasaki M, Fujiwara K, Harada S, Matsuda I, et al. Incidental bladder cancer found on cystoscopy during prostate biopsy: prevalence, pathological findings, and oncological outcome. Urol Int. 2022;106(8):791–7. 10.1159/000517895. 34352796

[13] Abufaraj M, Dalbagni G, Daneshmand S, Horenblas S, Kamat AM, Kanzaki R, et al. The role of surgery in metastatic bladder cancer: a systematic review. Eur Urol. 2018;73(4):543–57. 10.1016/j.eururo.2017.09.030. PMC8177016 29122377

[14] Zibelman M, Asghar AM, Parker DC, O’Neill J, Wei S, Greenberg RE, et al. Cystoscopy and systematic bladder tissue sampling in predicting pT0 bladder cancer: a prospective trial. J Urol. 2021;205(6):1605–11. 10.1097/JU.0000000000001602. PMC9006869 33535799

[15] Lavdaniti M, Zyga S. Quality of life in elderly bladder cancer patients following a cystectomy. Adv Exp Med Biol. 2017;989:297–300. 10.1007/978-3-319-57348-9_28. 28971439

[16] Novara G, Catto JW, Wilson T, Annerstedt M, Chan K, Murphy DG, et al. Systematic review and cumulative analysis of perioperative outcomes and complications after robot-assisted radical cystectomy. Eur Urol. 2015;67(3):376–401. 10.1016/j.eururo.2014.12.007. 25560798

[17] Ornaghi PI, Afferi L, Antonelli A, Cerruto MA, Odorizzi K, Gozzo A, et al. The impact of preoperative nutritional status on post-surgical complication and mortality rates in patients undergoing radical cystectomy for bladder cancer: a systematic review of the literature. World J Urol. 2021;39(4):1045–81. 10.1007/s00345-020-03291-z. 32519225

[18] Abe T, Yamada S, Kikuchi H, Sazawa A, Katano H, Suzuki H, et al. Impact of postoperative complications on long-term survival in bladder cancer patients. Jpn J Clin Oncol. 2023;53(10):966–76. 10.1093/jjco/hyad079. PMC10550200 37461191

[19] Haas M, Huber T, Pickl C, van Rhijn B, Guzvic M, Gierth M, et al. The comprehensive complication index is associated with a significant increase in complication severity between 30 and 90 days after radical cystectomy for bladder cancer. Ejso-Eur J Surg Oncol. 2021;47(5):1163–71. 10.1016/j.ejso.2020.09.040. 33046281

[20] Ren H, Tang P, Zhao Q, Ren G. Symptom clusters and related factors in bladder cancer patients three months after radical cystectomy. BMC Urol. 2017;17(1):65. 10.1186/s12894-017-0255-x. PMC5569499 28835243

[21] Tobert CM, Hamilton-Reeves JM, Norian LA, Hung C, Brooks NA, Holzbeierlein JM, et al. Emerging impact of malnutrition on surgical patients: literature review and potential implications for cystectomy in bladder cancer. J Urol. 2017;198(3):511–9. 10.1016/j.juro.2017.01.087. PMC5705177 28286066

[22] DiLizia EM, Sadeghi F. Surgical and pathological outcomes of robotic-assisted radical cystectomy for bladder cancer in the community setting. J Robot Surg. 2018;12(2):337–41. 10.1007/s11701-017-0740-y. 28836105

[23] Izquierdo L, Bolton DM, Lawrentschuk N. Radical cystectomy and orthotopic bladder substitution: surgical tricks and management of complications. Minerva Urol Nefrol. 2013;65(4):225–34. 24091476

[24] Abe T, Yamada S, Kikuchi H, Sazawa A. Impact of postoperative complications on long-term survival in bladder cancer patients. Jpn J Clin Oncol. 2023 Oct;53(10):966–76. 10.1093/jjco/hyad079. PMC10550200 37461191

[25] Weinberg L, Aitken SAA, Kaldas P. Postoperative complications and hospital costs following open radical cystectomy: A retrospective study. PLoS One. 2023 Feb;18(2):e0282324. 10.1371/journal.pone.0282324. PMC9956632 36827411

[26] Richters A, Ripping TM, Kiemeney LA, Leliveld AM, van Rhijn B, Oddens JR, et al. Hospital volume is associated with postoperative mortality after radical cystectomy for treatment of bladder cancer. BJU Int. 2021;128(4):511–8. 10.1111/bju.15334. PMC8519083 33404154

[27] Prcic A, Aganovic D, Hadziosmanovic O. Impact of complications and bladder cancer stage on quality of life in patients with different types of urinary diversions. Med Arch. 2013;67(6):418–22. 10.5455/medarh.2013.67.418-422. PMC4272483 25568512

[28] Arora A, Pugliesi F, Zugail AS, Moschini M, Pazeto C, Macek P, et al. Comparing perioperative complications between laparoscopic and robotic radical cystectomy for bladder cancer. J Endourol. 2020;34(10):1033–40. 10.1089/end.2020.0112. 32597214

[29] Liu H, Zhou Z, Yao H, Mao Q, Chu Y, Cui Y, et al. Robot-assisted radical cystectomy vs open radical cystectomy in patients with bladder cancer: a systematic review and meta-analysis of randomized controlled trials. World J Surg Oncol. 2023 Aug;21(1):240. 10.1186/s12957-023-03132-4. PMC10403906 37542288

[30] Zhou Y, Yang H, Liang Z. Efficiency and safety of laparoscopic radical cystectomy for muscle-invasive bladder cancer, and postoperative recurrence. Arch Esp Urol. 2023 May;76(3):196–202. 10.56434/j.arch.esp.urol.20237603.23. 37340525

[31] Zhong L, Wang F. Comparative analysis of wound healing techniques in postoperative bladder cancer patients. Int Wound J. 2024 Mar;21(3):e14820. 10.1111/iwj.14820. PMC10904970 38425151

